# Management of Clozapine Induced Hypersalivation on Slow Stream Rehabilitation Ward

**DOI:** 10.1192/bjo.2025.10447

**Published:** 2025-06-20

**Authors:** Neeti Sud, Yousra Ghandour

**Affiliations:** Cumbria Tyne and Wear NHS Foundation Trust, Morpeth, United Kingdom

## Abstract

**Aims:** Sialorrhoea (hypersalivation), a common side effect of clozapine can impact the quality of life of patients. At present, no drugs are licensed to manage clozapine-induced hypersalivation, but there are various practical and pharmacological management options included in literature. These include chewing sugarless gums during the day. At night, covering the pillow with a towel, elevating the head and sleeping on the side may reduce aspiration risk. With regard to pharmacological treatment, the first step should be to review the clozapine dose and reduce it if possible. The second step is to consider adding anticholinergic, antihistaminergic and adrenergic drugs and substitute benzamides such as amisulpride. There is also consideration of injecting botulism toxin to salivary glands.

**Methods:** We retrospectively audited the case notes of all six patients on clozapine in our male inpatient slow-stream rehabilitation service to assess if we have actively attempted to manage clozapine-induced hypersalivation side effects. We searched keywords ‘hypersalivation’, ‘drooling’, ‘pillow’, ‘saliva’ to identify case note entries and collected data on strategies used to manage hypersalivation. We reviewed past and current prescriptions and doses.

**Results:** Age ranges of our patients varied from 26 to 65. All six patients reported hypersalivation as a side effect. All patients had clozapine within therapeutic range with no option to reduce further. One patient preferred not to be on any medication to manage this side effect. All other patients had tried hyoscine hydrobromide tablets first. The tablet has a half-life of 4 hours. One patient was due a dose review of hyoscine due to ongoing hypersalivation. Three patients had been asked to suck or chew the tablets. Prior to this they had been swallowing the tablets. Two patients had tried the hyoscine patch (which lasts approximately 72 hours) but had found it not helpful. One patient had tried trihexyphenidyl tablets but then requested to change back to hyoscine. One patient had tried atropine drops following a trial of hyoscine tablets and patch. He then tried amisulpride with no impact and subsequently found trihexyphenidyl beneficial. All patients were monitored for worsened constipation with addition of anticholinergics.

**Conclusion:** The audit identified the need to proactively and systematically manage hypersalivation. It was also noted that practical interventions like raising the pillow, chewing gum during day were not routinely tried. We plan to re-audit the service in a year’s time to see if there is any improvement in use of management strategies and also measure hypersalivation using Nocturnal Hypersalivation Rating Scale and the Drooling Severity and Frequency Scale.

